# UFLC-PDA-MS/MS Profiling of Seven *Uncaria* Species Integrated with Melatonin/5-Hydroxytryptamine Receptors Agonistic Assay

**DOI:** 10.1007/s13659-020-00230-8

**Published:** 2020-01-13

**Authors:** Jian-Gang Zhang, Xiao-Yan Huang, Yun-Bao Ma, Ji-Jun Chen, Chang-An Geng

**Affiliations:** 1 State Key Laboratory of Phytochemistry and Plant Resources in West China, Kunming Institute of Botany, Chinese Academy of Sciences, Yunnan Key Laboratory of Natural Medicinal Chemistry, 132# Lanhei Road, Kunming, 650201 Yunnan People’s Republic of China; 2 University of Chinese Academy of Sciences, Beijing, 100049 People’s Republic of China

**Keywords:** Uncariae Ramulus Cum Uncis, *Uncaria* plants, LCMS-IT-TOF analyses, Melatonin and 5-hydroxytryptamine receptors

## Abstract

**Abstract:**

Uncariae Ramulus Cum Uncis (Gou-Teng), the dried hook-bearing stems of several *Uncaria* plants (Rubiaceae), is a well-known herbal medicine in China. The clinical application of Gou-Teng is bewildered for the morphological and chemical similarity between different species. In order to discern their chemical and biological difference, an ultra-fast liquid chromatography equipped with ion trap time-of-flight mass spectrometry (UFLC-IT/TOF-MS) combining with melatonin (MT_1_ and MT_2_) and 5-hydroxytryptamine (5-HT_1A_ and 5-HT_2C_) receptors agonistic assay in vitro was conducted on seven *Uncaria* species. As a result, 57 compounds including 35 indole alkaloids, ten flavonoids, five triterpenoids, five chlorogenic analogues, and two other compounds were characterized based on their MS/MS patterns and UV absorptions. Specifically, cadambine-type and corynanthein-type alkaloids were exclusively present in *U.rhynchophylla* and *U.scandens*, whereas corynoxine-type alkaloids were commonly detected in all the seven *Uncaria* plants. Three *Uncaria* species, *U. rhynchophylla*, *U. macrophylla*, and *U. yunnanensis* showed obviously agnostic activity on four neurotransmitter receptors (MT_1_, MT_2_, 5-HT_1A_, and 5-HT_2C_). This first-time UFLCMS-IT-TOF analyses integrated with biological assay on seven *Uncaria* plants will provide scientific viewpoints for the clinical application of Gou-Teng.

**Graphic Abstract:**

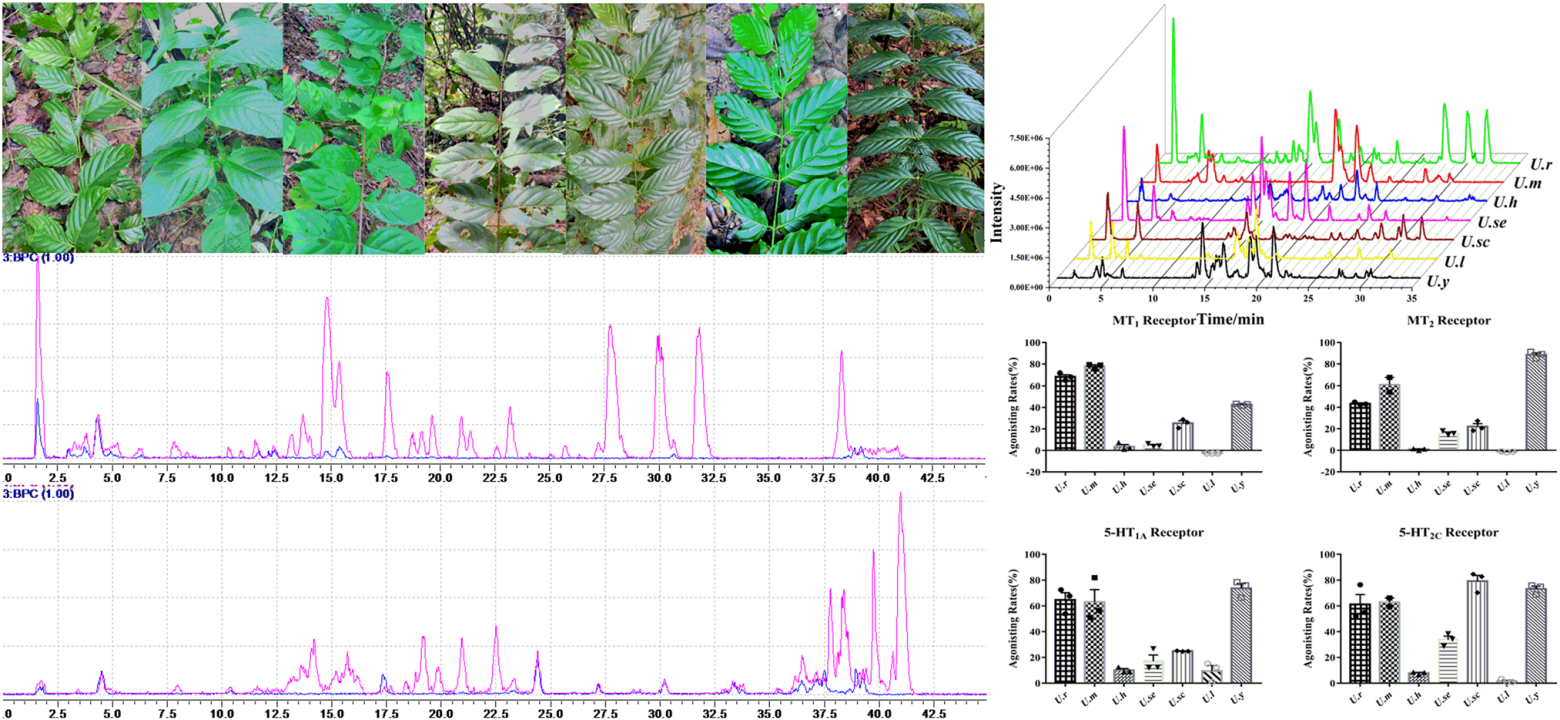

**Electronic supplementary material:**

The online version of this article (10.1007/s13659-020-00230-8) contains supplementary material, which is available to authorized users.

## Introduction

Uncariae Ramulus Cum Uncis (Gou-Teng), the dried hook-bearing stems of *Uncaria* plants (Rubiaceae), is a well-known traditional Chinese medicine (TCM), which has long been used for the treatment of hypertension, fever, headache, dizziness, stroke, and bilious disorders in China [1–4]. In addition to monotherapies, Gou-Teng is also prescribed in many formulae, such as Diao-Teng San (Cho-Deung-San in Korean and Choto-san in Japanese) and Yi-Gan San (Yokukansan in Japanese) [2]. Indole alkaloids as the characteristic constituents of *Uncaria* plants are responsible for the hypotensive effects, *e.g.* rhynchophylline and hirsutine showing antihypertensive and antiarrhythmic effects [5, 6]. According to the latest Chinese Pharmacopoeia (2015 edition), five *Uncaria* plants, namely *Uncaria rhynchophylla* (*U. r*), *Uncaria macrophylla* (*U. m*), *Uncaria sinensis* (*U. si*), *Uncaria hirsuta* (*U. h*), and *Uncaria sessilifructus* (*U. se*), are documented as the official resource of Gou-Teng [7]. Furthermore, several *Uncaria* plants, *e.g. Uncaria scandens* (*U. sc*), *Uncaria laevigata* (*U. l*), and *Uncaria yunnanensis* (*U. y*), are also used as the substitutes of Gou-Teng in prescriptions [8, 9]. Although recent studies have manifested the antidepressant-like effects of *U. rhynchophylla* and *U. lanosa*, and locomotor decreasing effects of *U. rhynchophylla*, *U. macrophylla*, and *U. sinensis* [10–12], few reports can discern the difference regarding the chemical profiles and biological activities between different *Uncaria* species. Thus, the clinical application of Gou-Teng is bewildered for the morphological and chemical similarity between different *Uncaria* plants. Different from the cardiovascular effect, the psychiatric property and active constituents of Gou-Teng are still disputed. Melatonin (MT) and 5-hydroxytryptamine (5-HT) receptors are two types of neurotransmitter receptors closely related to mental diseases [13–16], and thus are used to evaluate the psychiatric effects of different *Uncaria* plants. The present study applied an ultra-fast liquid chromatography equipped with ion trap time-of-flight mass spectrometry (UFLC-IT/TOF-MS) and combined with melatonin and 5-hydroxytryptamine receptors agonistic assay to discern seven *Uncaria* species regarding their chemical profiles and psychiatric properties.

## Results and Discussions

### LCMS-PDA Analyses

**Fig. 1:**
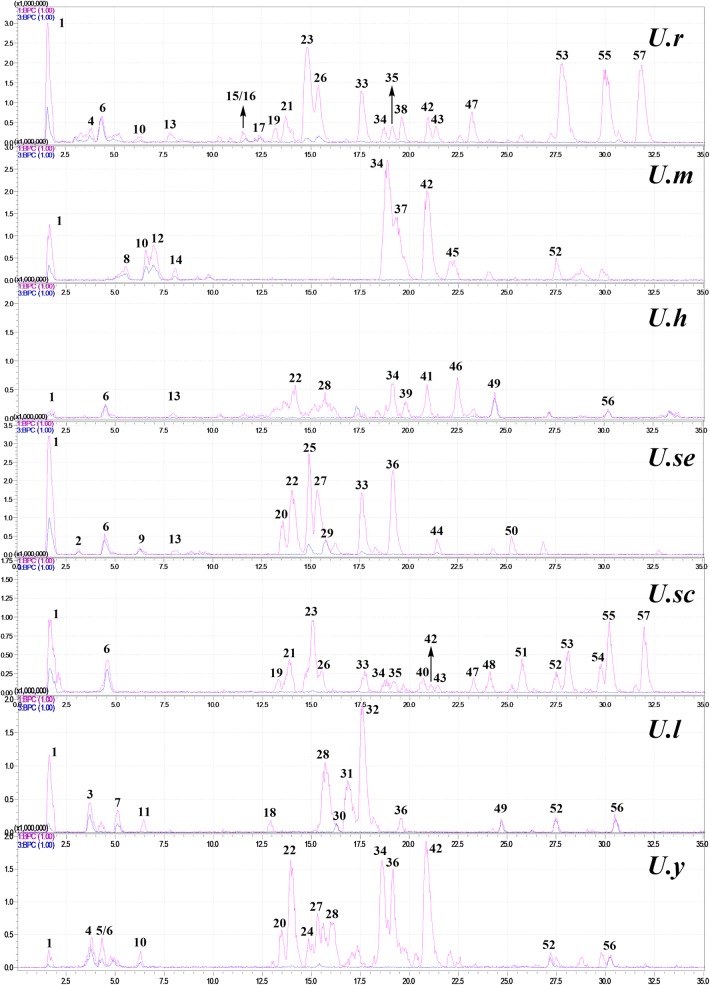
Base peak chromatograms (BPCs) of seven *Uncaria* plants in positive (1 BPC) and negative (3 BPC) modes

Seven *Uncaria* plants were analyzed by UFLC-PDA-MS/MS to provide their respective base peak chromatograms (BPCs) in both positive and negative modes (Fig. 1). In total, 57 compounds including 35 indole alkaloids, ten flavonoids, five triterpenoids, five chlorogenic acids, and two other compounds were characterized according to their UV absorptions, MS/MS fragmentations, retention time, and comparing with the reported compounds (Table 1).

**Table 1:** Characterization of peaks in seven *Uncaria* plants by UFLC-DAD-MS/MS analyses

No.	t_R_ (min)	MW	MF	DBE	MS	MS/MS	λ_max_ (nm)	Name
**1**	1.59	342	C_12_H_22_O_11_	2	Pos: 381.0792 ([M+K]^+^, − 0.2 mDa) Neg: 387.1170 ([M+HCOO]^−^, +2.6 mDa)	Pos: − Neg: 387 → 341.1091 (C_12_H_22_O_11_)	201	Sucrose
**2**	3.06	376	C_16_H_24_O_10_	5	Pos: 399.1258 ([M+Na]^+^, − 0.4 mDa) Neg: 375.1301 ([M−H]^−^, + 0.4 mDa)	Pos: 399 → 377.1439(C_16_H_24_O_10_), 215.0678 (C_13_H_10_O_3_) Neg: −	234	Loganic acid
**3**	3.71	354	C_16_H_18_O_9_	8	Pos: 355.1017 ([M+H]^+^, − 0.7 mDa) Neg: 353.0877 ([M−H]^−^, − 0.1 mDa)	Pos: 355 → 163.0406 (C_9_H_6_O_3_), 145.0335 (C_9_H_4_O_2_) Neg: 353 → 191.0565 (C_7_H_12_O_6_)	221 243 325	Neochlorogenic acid
**4**	3.80	578	C_30_H_26_O_12_	18	Pos: 579.1465 ([M+H]^+^, − 3.2 mDa) Neg: 577.1327 ([M−H]^−^, − 2.5 mDa)	Pos: 579 → 409.0915 (C_22_H_16_O_8_), 301.0701 (C_16_H_12_O_6_), 287.0553 (C_15_H_10_O_6_), 259.0128, 247.0453 Neg: 577 → 425.0872 (C_22_H_18_O_9_), 407.0766 (C_22_H_16_O_8_), 285.0352, 245.0817	279	Procyanidin B1
**5**	4.31	290	C_15_H_14_O_6_	9	Pos: 291.0841 ([M+H]^+^, − 2.2 mDa) Neg: 289.0712 ([M−H]^−^, − 0.6 mDa)	Pos: 291 → 273.0741 (C_15_H_12_O_5_), 139.0423 (C_7_H_6_O_3_), 123.0342 (C_7_H_6_O_2_) Neg: −	280	Catechin
**6**	4.58	354	C_16_H_18_O_9_	8	Pos: 355.1016 ([M+H]^+^, − 0.8 mDa) Neg: 353.0873 ([M−H]^−^, − 0.5 mDa)	Pos: 355 → 163.0407 (C_9_H_6_O_3_), 145.0254(C_9_H_4_O_2_) Neg: 353 → 191.0569 (C_7_H_12_O_6_)	218 234 325	Chlorogenic acid
**7**	5.10	354	C_16_H_18_O_9_	8	Pos: 355.1023 ([M+H]^+^, − 0.1 mDa) Neg: 353.0887 ([M−H]^−^, + 0.9 mDa)	Pos: 355 → 163.0401 (C_9_H_6_O_3_) Neg: 353 → 191.0565 (C_7_H_12_O_6_)	218 234 325	Cryptochlorogenic acid
**8**	5.57	578	C_30_H_26_O_12_	18	Pos: 579.1480 ([M+H]^+^, − 1.7 mDa) Neg: 577.1330 ([M−H]^−^, − 2.2 mDa)	Pos: 579 → 427.1024 (C_22_H_18_O_9_), 409.0924 (C_22_H_16_O_8_), 301.0766 (C_16_H_12_O_6_), 287.0693 (C_15_H_14_O_6_) Neg: 577 → 425.0911 (C_22_H_18_O_9_), 407.0742 (C_22_H_16_O_8_)	280	Procyanidin B2
**9**	6.22	354	C_16_H_18_O_9_	8	Pos: 355.1013 ([M+H]^+^, − 1.1 mDa) Neg: 353.0870 ([M−H]^−^, − 0.8 mDa)	Pos: 355 → 163.0420 (C_9_H_6_O_3_) Neg: 353 → 191.0573 (C_7_H_12_O_6_)	218 234 325	Isochlorogenic acid
**10**	6.28	290	C_15_H_14_O_6_	9	Pos: 291.0841 ([M+H]^+^, − 2.2 mDa) Neg: 289.0704 ([M−H]^−^, − 1.4 mDa)	Pos: 291 → 139.0411 (C_7_H_6_O_3_), 123.0342 (C_7_H_6_O_2_) Neg: −	280	Epicatechin
**11**	6.43	370	C_21_H_26_N_2_O_4_	10	Pos: 371.1973 ([M+H]^+^, + 0.8 mDa) Neg: −	Pos:371 → 353.1889 (C_21_H_24_N_2_O_3_), 267.1463 (C_17_H_18_N_2_O), 229.1376 (C_14_H_16_N_2_O) Neg: −	241	Corynoxinic acid
**12**	7.00	562	C_30_H_26_O_11_	18	Pos: 563.1514 ([M+H]^+^, − 3.4 mDa) Neg: 561.1392 ([M−H]^−^, − 1.0 mDa)	Pos: 563 → 411.1049 (C_22_H_18_O_8_), 393.0997 (C_22_H_16_O_7_), 291.0856 (C_15_H_14_O_6_), 273.0778 (C_15_H_12_O_5_) Neg: 561 → 407.0755 (C_22_H_16_O_8_), 289.0693 (C_15_H_14_O_6_), 187.0425	275	Fisetinidol-(4*α* → 8)- epicatechin
**13**	7.87	468	C_21_H_24_O_12_	10	Pos: 469.1323 ([M+H]^+^, − 1.8 mDa) Neg: −	Pos: 469 → 317.0994 (C_16_H_12_O_7_) Neg: −	278	Gallocatechol *C*-glucoside
**14**	8.09	562	C_30_H_26_O_11_	18	Pos: 563.1526 ([M+H]^+^, − 2.2 mDa) Neg: 561.1400 ([M−H]^−^, − 0.2 mDa)	Pos: 563 → 411.1014 (C_22_H_18_O_8_), 393.0943 (C_22_H_16_O_7_), 291.0822 (C_15_H_14_O_6_), 287.0646 (C_15_H_10_O_6_), 267.0542, 231.0657 Neg: 561 → 407.0783 (C_22_H_16_O_8_), 289.0707 (C_15_H_14_O_6_)	277	Fisetinidol-(4*β* → 8)- epicatechin
**15**	11.59	838	C_38_H_50_N_2_O_19_	15	Pos: 839.3054 ([M+H]^+^, − 2.7 mDa) Neg: 883.3029([M+HCOO]^−^, + 3.9 mDa)	Pos:839 → 677.2546 (C_32_H_40_N_2_O_14_), 515.1975 (C_26_H_30_N_2_O_9_), 353.1502 (C_20_H_20_N_2_O_4_), 283.1141 (C_16_H_14_N_2_O_3_) Neg: 883 → 837.2849 (C_38_H_50_N_2_O_19_), 675.2278 (C_32_H_40_N_2_O_14_), 495.1688(C_26_H_28_N_2_O_8_), 281.0865 (C_16_H_14_N_2_O_3_)	283	Vincosamide 11,6′-di-*O*-*β*-d- glucopyranoside
**16**	11.66	610	C_27_H_30_O_16_	13	Pos: 611.1585 ([M+H]^+^, − 2.2 mDa) Neg: 609.1454 ([M−H]^−^, − 0.7 mDa)	Pos:611 → 303.0473 (C_15_H_10_O_7_) Neg: 609 → 301.0321 (C_15_H_10_O_7_), 255.0311 (C_14_H_8_O_5_)	253 348	Rutin
**17**	12.40	464	C_21_H_20_O_12_	12	Pos: 465.1019 ([M+H]^+^, − 0.9 mDa) Neg: 463.0871 ([M−H]^−^, − 1.1 mDa)	Pos: 465 → 303.0497 (C_15_H_10_O_7_) Neg: 463 → 301.0300 (C_15_H_10_O_7_), 271.0146 (C_14_H_8_O_6_)	255 354	Hyperoside
**18**	12.92	516	C_26_H_32_N_2_O_9_	12	Pos:517.2206 ([M+H]^+^, + 2.5 mDa) Neg: −	Pos: 517 → 338.1546 (C_20_H_19_NO_4_), 276.1250 (C_19_H_17_NO) Neg: −	203 280	Strictosidinic acid
**19**	13.24	564	C_27_H_36_N_2_O_11_	11	Pos: 565.2385 ([M+H]^+^, − 0.7 mDa) Neg: −	Pos: 565 → 548.2101 (C_27_H_33_NO_11_), 386.1677 (C_21_H_23_NO_6_), 354.1487 (C_20_H_19_NO_5_) Neg: −	220 279	Hydrated cadambine
**20**	13.55	368	C_21_H_24_N_2_O_4_	11	Pos: 369.1801 ([M+H]^+^, − 0.8 mDa) Neg: −	Pos:369 → 337.1568 (C_20_H_20_N_2_O_3_), 267.1447 (C_17_H_18_N_2_O), 241.1439 (C_15_H_16_N_2_O), 213,1067 (C_13_H_12_N_2_O), 160.0747 (C_10_H_9_NO) Neg: −	205 240	Cisocorynoxeine
**21**	13.86	544	C_27_H_32_N_2_O_10_	13	Pos: 545.2108 ([M+H]^+^, − 2.2 mDa) Neg: −	Pos: 545 → 383.1612 (C_21_H_22_N_2_O_5_), 351.1245 (C_20_H_18_N_2_O_4_), 263.1091 (C_16_H_10_N_2_O_2_), 227.1193(C_14_H_14_N_2_O) Neg: −	280	Cadambine
**22**	14.08	368	C_21_H_24_N_2_O_4_	11	Pos: 369.1800 ([M+H]^+^, − 0.9 mDa) Neg: −	Pos:369 → 337.1590 (C_20_H_20_N_2_O_3_), 291.1455 (C_19_H_18_N_2_O), 265.1246 (C_17_H_16_N_2_O), 213,0997 (C_13_H_12_N_2_O), 160.0682 (C_10_H_9_NO) Neg: −	206 241	18,19-Dehydrocorynoxinic acid
**23**	14.81	546	C_27_H_34_N_2_O_10_	12	Pos: 547.2269 ([M+H]^+^, − 1.7 mDa) Neg: 591.2205 ([M+HCOO]^−^, + 1.0 mDa)	Pos:547 → 385.1801 (C_21_H_24_N_2_O_5_), 367.1688 (C_21_H_22_N_2_O_4_), 349.1577 (C_21_H_20_N_2_O_3_), 335.1372 (C_20_H_18_N_2_O_3_), 317.1258 (C_20_H_16_N_2_O_2_) Neg: 591 → 545.2097 (C_27_H_34_N_2_O_10_)	220 278	3*α*-Dihydrocadambine
**24**	14.86	382	C_22_H_26_N_2_O_4_	11	Pos: 383.1955 ([M+H]^+^, − 1.0 mDa) Neg: −	Pos:383 → 351.1743 (C_21_H_22_N_2_O_3_), 241.1262 (C_15_H_16_N_2_O) Neg: −	205 244	Isocorynoxeine
**25**	14.90	570	C_28_H_30_N_2_O_11_	15	Pos:571.1896 ([M+H]^+^, − 2.6 mDa) Neg: 569.1780 ([M−H]^−^, + 0.3 mDa)	Pos: 571 → 409.1426 (C_22_H_20_N_2_O_6_), 391.1250 (C_22_H_18_N_2_O_5_), 377.1120 (C_21_H_16_N_2_O_5_), 359.1064 (C_21_H_14_N_2_O_4_), 341.0952 (C_21_H_12_N_2_O_3_), 313.0973 (C_20_H_12_N_2_O_2_) Neg: 569 → 389.1187 (C_22_H_18_N_2_O_5_)	216 238 275 374	Deoxycordifoline
**26**	15.33	546	C_27_H_34_N_2_O_10_	12	Pos: 547.2252 ([M+H]^+^, − 3.4 mDa) Neg: 591.2193 ([M+HCOO]^−^, − 0.2 mDa)	Pos:547 → 385.1762 (C_21_H_24_N_2_O_5_), 367.1697 (C_21_H_22_N_2_O_4_), 353.1595 (C_20_H_20_N_2_O_4_), 335.1441 (C_20_H_18_N_2_O_3_) Neg: −	218 280	3*β*-Dihydrocadambine
**27**	15.38	384	C_21_H_24_N_2_O_5_	11	Pos: 385.1739 ([M+H]^+^, − 1.9 mDa) Neg: 429.1682 ([M+HCOO]^−^, + 1.5 mDa)	Pos:385 → 367.1698 (C_21_H_22_N_2_O_4_), 351.1696 (C_21_H_22_N_2_O_3_), 335.1338 (C_20_H_18_N_2_O_3_), 267.1399 (C_17_H_18_N_2_O), 239.1202 (C_15_H_14_N_2_O) Neg: −	206 240	Oxocorynoxinic acid
**28**	15.69	368	C_21_H_24_N_2_O_4_	11	Pos: 369.1811 ([M+H]^+^, + 0.2 mDa) Neg: −	Pos:369 → 337.1588 (C_20_H_20_N_2_O_3_), 309.1647 (C_19_H_20_N_2_O_2_), 291.1455 (C_19_H_18_N_2_O), 265.1246 (C_17_H_16_N_2_O), 160.0810 (C_10_H_9_NO) Neg: −	204 240	18,19-Dehydrocorynoxinic acid B
**29**	15.74	448	C_21_H_20_O_11_	12	Pos: 449.1068 ([M+H]^+^, − 1.0 mDa) Neg: 447.0939 ([M−H]^−^, + 0.6 mDa)	Pos: 449 → 303.0510 (C_15_H_10_O_7_) Neg: 447 → 301.0358 (C_15_H_10_O_7_), 271.0288 (C_14_H_8_O_6_)	204 255 348	Quercetin 3-rhamnoside
**30**	16.28	516	C_25_H_24_O_12_	14	Pos: 517.1337 ([M+H]^+^, − 0.4 mDa) Neg: 515.1187 ([M−H]^−^, − 0.8 mDa)	Pos: − Neg: 515 → 353.0882 (C_16_H_18_O_9_), 173.0401 (C_7_H_10_O_5_)	247 326	3,5-Dicaffeoylquinic acid
**31**	16.89	384	C_21_H_24_N_2_O_5_	11	Pos: 385.1756 ([M+H]^+^, − 0.2 mDa) Neg: 429.1686 ([M+HCOO]^−^, + 1.9 mDa)	Pos:385 → 367.1671 (C_21_H_22_N_2_O_4_), 351.1721 (C_21_H_22_N_2_O_3_), 223.1210 (C_15_H_14_N_2_) Neg: −	249	Oxorhynchophyllic acid
**32**	17.59	368	C_21_H_24_N_2_O_4_	11	Pos: 369.1817 ([M+H]^+^, + 0.8 mDa) Neg: −	Pos: 369 → 337.1650 (C_20_H_20_N_2_O_3_), 309.1566 (C_19_H_20_N_2_O_2_), 241.1312 (C_15_H_16_N_2_O), 187.0844 (C_11_H_10_N_2_O), 160.0773 (C_10_H_9_NO) Neg: −	246	Demethylcorynoxeine
**33**	17.63	530	C_27_H_34_N_2_O_9_	12	Pos: 531.2308 ([M+H]^+^, − 2.9 mDa) Neg: 575.2225 ([M+HCOO]^−^, − 2.1 mDa)	Pos:531 → 514.2026 (C_27_H_31_NO_9_), 352.1577 (C_21_H_21_NO_4_), 334.1493 (C_21_H_19_NO_3_) Neg: −	220 279	3-Epistrictosidine
**34**	18.62	384	C_22_H_28_N_2_O_4_	10	Pos: 385.2112 ([M+H]^+^, − 1.0 mDa) Neg: −	Pos: 385 → 353.1852 (C_21_H_24_N_2_O_3_), 321.1618 (C_20_H_20_N_2_O_2_), 267.1494 (C_17_H_18_N_2_O), 241.1331 (C_15_H_16_N_2_O), 187.0798 (C_11_H_10_N_2_O) Neg: −	206 243	Isorhynchophylline
**35**	19.16	382	C_22_H_26_N_2_O_4_	11	Pos: 383.1959 ([M+H]^+^, − 0.6 mDa) Neg: −	Pos:383 → 351.1671 (C_21_H_22_N_2_O_3_), 319.1480 (C_20_H_18_N_2_O_2_), 267.1530 (C_17_H_18_N_2_O), 215.1098 (C_13_H_14_N_2_O), 160.0682 (C_10_H_9_NO) Neg: −	202 240	Corynoxeine
**36**	19.21	368	C_21_H_24_N_2_O_4_	11	Pos: 369.1783 ([M+H]^+^, − 2.6 mDa) Neg: −	Pos:369 → 337.1539 (C_20_H_20_N_2_O_3_), 293.1347 (C_18_H_16_N_2_O_2_), 267.1553 (C_17_H_18_N_2_O), 239.1232 (C_15_H_14_N_2_O), 160.0760 (C_10_H_9_NO) Neg: −	207 242	Demethylisocorynoxeine
**37**	19.32	384	C_22_H_28_N_2_O_4_	10	Pos: 385.2133 ([M+H]^+^, + 1.1 mDa) Neg: −	Pos:385 → 353.1875 (C_21_H_24_N_2_O_3_), 321.1619 (C_20_H_20_N_2_O_2_), 265.1339 (C_17_H_16_N_2_O), 241.1334 (C_15_H_16_N_2_O), 187.0697 (C_11_H_10_N_2_O) Neg: −	210 243	Corynoxine
**38**	19.62	530	C_27_H_34_N_2_O_9_	12	Pos: 531.2311 ([M+H]^+^, − 2.6 mDa) Neg: 575.2231 ([M+HCOO]^−^, − 1.5 mDa)	Pos:531 → 514.2082 (C_27_H_31_NO_9_), 352.1586 (C_21_H_21_NO_4_), 334.1556 (C_21_H_19_NO_3_) Neg: −	219 280	Strictosidine
**39**	19.89	352	C_21_H_24_N_2_O_3_	11	Pos: 353.1854 ([M+H]^+^, − 0.6 mDa) Neg: −	Pos:353 → 321.1647 (C_20_H_20_N_2_O_2_), 222.1198 (C_12_H_15_NO_3_), 210.1126 (C_11_H_15_NO_3_), 144.0798 (C_10_H_9_N) Neg: −	219 280	Ajmalicine
**40**	20.63	354	C_21_H_26_N_2_O_3_	10	Pos: 355.1994 ([M+H]^+^, − 2.2 mDa) Neg: 353.1879 ([M−H]^−^, + 0.8 mDa)	Pos: 354 → 224.1340 (C_12_H_17_NO_3_), 212.1241 (C_11_H_17_NO_3_), 144.0792 (C_10_H_9_N) Neg: −	220 281	Sitsirikine
**41**	20.96	580	C_31_H_48_O_10_	8	Pos: 581.3333 ([M+H]^+^, + 1.3 mDa) Neg:579.3123 ([M−H]^−^, − 1.2 mDa)	Pos: 581 → 389.2065 (C_26_H_28_O_3_) Neg: −	202	Demythyl atropuroside C
**42**	20.96	384	C_22_H_28_N_2_O_4_	10	Pos: 385.2101 ([M+H]^+^, − 2.1 mDa) Neg: −	Pos: 385 → 353.1847 (C_21_H_24_N_2_O_3_), 321.1589 (C_20_H_20_N_2_O_2_), 267.1539 (C_17_H_18_N_2_O), 265.1373 (C_17_H_16_N_2_O), 160.0632 (C_10_H_9_NO) Neg: −	210 242	Rhynchophylline
**43**	21.37	352	C_21_H_24_N_2_O_3_	11	Pos: 353.1829 ([M+H]^+^, − 3.1 mDa) Neg: 351.1726 ([M−H]^−^, + 1.2 mDa)	Pos: 353 → 304.1399 (C_20_H_17_NO_2_), 222.1162 (C_12_H_15_NO_3_), 210.1111 (C_11_H_15_NO_3_), 144.0861 (C_10_H_9_N) Neg: −	219 278	Geissoschizine
**44**	21.48	930	C_44_H_54_N_2_O_20_	19	Pos: 931.3357 ([M+H]^+^, + 1.4 mDa) Neg:929.3174 ([M−H]^−^, − 2.3 mDa)	Pos: 931 → 769.2802 (C_38_H_44_N_2_O_15_), 719.2172(C_37_H_38_N_2_O_13_), 607.2281 (C_32_H_34_N_2_O_10_), 557.1858 Neg: 929 → 749.2512 (C_38_H_42_N_2_O_14_), 517.1466 (C_24_H_26_N_2_O_11_)	219	Neonaucleoside C
**45**	22.23	400	C_22_H_28_N_2_O_5_	10	Pos: 401.2090 ([M+H]^+^, + 1.9 mDa) Neg: −	Pos: 401 → 383.1953 (C_22_H_26_N_2_O_4_), 355.1652 (C_20_H_22_N_2_O_4_), 241.1699 (C_16_H_20_N_2_), 239.1543 (C_16_H_18_N_2_) Neg: −	212 280	Dihydroxycorynantheine
**46**	22.52	594	C_32_H_50_O_10_	8	Pos: 595.3477 ([M+H]^+^, − 3.1 mDa) Neg:593.3207 ([M−H]^−^, + 2.8 mDa)	Pos: 595 → 567.3522 (C_31_H_50_O_9_), 536.2769 (C_32_H_40_O_7_), 389.2051 (C_26_H_28_O_3_) Neg: −	204	Atropuroside C
**47**	23.19	546	C_27_H_34_N_2_O_10_	12	Pos: 547.2286 ([M+H]^+^, + 3.8 mDa) Neg: 591.2195 ([M+HCOO]^−^, + 2.2 mDa)	Pos:547 → 385.1740 (C_21_H_24_N_2_O_5_), 367.1648(C_21_H_22_N_2_O_4_), 349.1520 (C_21_H_20_N_2_O_3_), 335.1317 (C_20_H_18_N_2_O_3_) Neg: 591 → 383.1612 (C_21_H_24_N_2_O_5_)	202 217 279	3*β*-Isodihydrocadambine
**48**	24.10	366	C_22_H_26_N_2_O_3_	11	Pos: 367.2016 ([M+H]^+^, − 2.1 mDa) Neg: −	Pos:367 → 251.1628 (C_17_H_18_N_2_), 236.1268 (C_13_H_17_NO_3_), 224.1199 (C_12_H_17_NO_3_), 192.1019 (C_11_H_13_NO_2_) Neg: −	220 280	Corynantheine
**49**	24.40	810	C_42_H_66_O_15_	10	Pos: 833.4294 ([M+Na]^+^, − 3.6 mDa), 469.3306 (C_30_H_44_O_4_) Neg: 809.4329 ([M−H]^−^, − 2.5 mDa)	Pos:469 → 451.3204 (C_30_H_42_O_3_), 423.3278 (C_29_H_42_O_2_), 379.3331 (C_28_H_42_), 263.1778 (C_20_H_22_) Neg: 809 → 603.3873 (C_35_H_56_O_8_)	207	Quinovic acid diglycoside
**50**	25.25	902	C_44_H_58_N_2_O_18_	17	Pos: 903.3714 ([M+H]^+^, − 4.3 mDa) Neg: 901.3615 ([M−H]^−^, + 0.3 mDa)	Pos: 903 → 341.1434 (C_19_H_20_N_2_O_4_), 323.1406 (C_19_H_18_N_2_O_3_) Neg: −	221 280	Bahienoside B
**51**	25.70	368	C_22_H_28_N_2_O_3_	10	Pos: 369.2154 ([M+H]^+^, + 2.6 mDa) Neg: −	Pos:369 → 251.1179 (C_14_H_22_N_2_O_2_), 238.1458 (C_13_H_19_NO_3_), 226.1418 (C_12_H_19_NO_3_) Neg: −	220	Dihydrocorynantheine
**52**	27.51	956	C_48_H_76_O_19_	11	Pos: 979.4895 ([M+Na]^+^, + 2.2 mDa) Neg: 955.4917 ([M−H]^−^, + 0.9 mDa)	Pos:979 → 935.434 (C_47_H_76_O_17_), 773.4421 Neg: 955 → 749.4438 (C_41_H_66_O_12_), 587.3923 (C_35_H_56_O_7_), 441.3496	204	Quinovic acid triglycoside
**53**	27.74	366	C_22_H_26_N_2_O_3_	11	Pos: 367.2011 ([M+H]^+^, − 0.5 mDa) Neg: −	Pos: 367 → 249.1363 (C_17_H_16_N_2_) Neg: −	221 280	Geissoschizine methyl ether
**54**	29.76	382	C_22_H_26_N_2_O_4_	11	Pos: 383.1932 ([M+H]^+^, − 3.3 mDa) Neg: −	Pos:367 → 223.1304 (C_15_H_14_N_2_), 184.0878 (C_12_H_9_NO) Neg: −	206 224 348	Pubescin
**55**	30.02	366	C_22_H_26_N_2_O_3_	11	Pos: 367.2007 ([M+H]^+^, − 0.9 mDa) Neg: −	Pos:367 → 251.1606 (C_17_H_18_N_2_), 224.1386 (C_16_H_17_N) Neg: −	221 280	Hirsuteine
**56**	30.52	486	C_30_H_46_O_5_	8	Pos: 487.3404 ([M+H]^+^, − 1.4 mDa) Neg: −	Pos: 469 → 451.3117 (C_30_H_42_O_3_), 423.3082 (C_29_H_42_O_2_) Neg: −	202	Quinovic acid
**57**	31.84	368	C_22_H_28_N_2_O_3_	10	Pos: 369.2154 ([M+H]^+^, − 1.9 mDa) Neg: −	Pos: 369 → 337.1945 (C_21_H_24_N_2_O_2_), 238.1481 (C_13_H_19_NO_3_), 226.1380 (C_12_H_19_NO_3_) Neg: −	221 280	Hirsutine

#### Indole Alkaloids

Indole alkaloids are the characteristic constituents in *Uncaria* plants with high response in positive mode MS. In this investigation, a number of 35 indole alkaloids were described and divided into six subclasses including cadambine-type (**19**, **21**, **23**, **26**, **47**), vinsosamide-type (**15**), D-*seco*-type (**18**, **25**, **33**, **38**, **44**, **50**), corynoxine-type (**11**, **20**, **22**, **24**, **27**, **28**, **31**, **32**, **34**, **35**, **36**, **37**, **42**), corynanthein-type (**40**, **43**, **45**, **48**, **51**, **53**, **55**, **57**), and ajmalicine-type (**39**, **54**). In accordance with the previous investigation [17], D-*seco* alkaloids commonly generated the characteristic fragmentation ions ascribed to the loss of 17 Da (NH_3_) in the MS^2^ experiment; the indole and oxindole alkaloids could be differentiated from their respective maximal UV absorptions around 280 nm (indole) or 240 nm (oxindole); the numbers and types of glycosyl moieties were determined by the mass defects between the parent and fragment ions.

##### Cadambine-Type Alkaloids

Peak **21** was identified as cadambine from the [M+H]^+^ ion at *m/z* 545.2129 with the diagnostic MS^2^ ions at *m*/*z* 383.1612 (C_21_H_22_N_2_O_5_) and 351.1245 (C_20_H_18_N_2_O_4_), corresponding to the sequential loss of glycosyl and MeOH moieties [18]. Peak **19** showed the loss of 17 Da from 565 to 548, and the loss of 162 Da from 548 to 386, which was characteristic for the hydrated derivative of cadambine [18]. Peaks **23**, **26**, and **47** possessed the same molecular formula of C_27_H_34_N_2_O_10_ with two more hydrogens than **21**. In the MS^2^ spectra, the identical fragmentation at *m*/*z* 385 (C_21_H_24_N_2_O_5_) and 367 (C_21_H_22_N_2_O_4_) suggested closely related structures. In accordance with the previous reports, 3*α*-dihydrocadambine, 3*β*-dihydrocadambine, and 3*β*-isodihydrocadambine were reasonably suggested [19].

##### Vincosamide-Type Alkaloids

Peak **15** showing a molecular formula of C_38_H_50_N_2_O_19_ was deduced from the [M+H]^+^ ion at *m*/*z* 839.3054. In the positive MS^2^ experiment, the sequential losses of three glycosyl moieties (C_6_H_10_O_5_, 162 Da) suggested the presence of three glucosyl in the structure. Finally, this compound was isolated under the guidance of LCMS analysis, and identified to be 2′-*O*-[*β*-d-glucopyranosyl-(1 → 6)-*β*-d-glucopyranosyl]-11-hydroxyvincosamide based on rigid 1D and 2D NMR spectroscopic data [20].

##### D-seco Indole Alkaloids

D-*seco* indole alkaloids can be well recognized from the diagnostic MS^2^ ions attributed to the neutral loss of 17 Da (NH_3_) from the precursor ions. Peaks **33** and **38** were assigned with the same molecular formula of C_27_H_34_N_2_O_9_ from the [M+H]^+^ ion at *m*/*z* 531. Their similar MS^2^ fragmentations at *m*/*z* 514 (C_27_H_31_NO_9_) and 352 (C_21_H_21_NO_4_) indicated a pair of isomers, which were generated from the cleavage of 3-*epi-*strictosidine and strictosidine [21]. Peak **18** with a molecular weight of 516 was deduced to be the demethylated derivative of **38**, owing to a CH_2_ (14 Da) less in the molecular formula. The MS^2^ fragmentation ion at *m*/*z* 338.1568 implied the successive loss of 17 Da (NH_3_) and 162 Da (C_6_H_10_O_5_), by which this compound was assigned as strictosidinic acid [22]. The molecular formula of **25** was determined as C_28_H_30_N_2_O_11_ by the protonated ion ([M+H]^+^) at *m*/*z* at 571.1896 and deprotonated ion ([M‒H]^‒^) at *m/z* 569.1780. In the MS^2^ experiment, the sequential losses of 162 Da (C_6_H_10_O_5_), 18 Da (H_2_O), and 14 Da (CH_2_) was consistent with the presence of glucosyl, hydroxyl, and methoxyl groups. From the above analyses, peak **25** was tentatively assigned as desoxycordifoline that had been isolated from *Chimarrhis turbinate* [23]. Peaks **44** and **50** shared the molecular weight of *m/z* 930 and 902, respectively, corresponding to the chemical composition of C_44_H_54_N_2_O_20_ and C_44_H_58_N_2_O_18_. The sequential losses of two 162 Da (C_6_H_10_O_5_) indicated the presence of two glucosyls. Taking its UV absorption at 219 nm into consideration, peak **44** was tentatively deduced to be neonaucleoside C [24]. Similarly, peak **50** was attributed to be bahienoside B from the fragments at *m/z* 341.1434 (C_19_H_20_N_2_O_4_) and 323.1406 (C_19_H_18_N_2_O_3_), by retrieving the compounds isolated from the same genus [25].

##### Corynoxine-Type Alkaloids

The spirocyclic corynoxine-type alkaloids account for the largest number of indole alkaloids within *Uncaria* genus. Generally, this type of alkaloids can be well recognized by their UV maximum absorption at about 240 nm [17]. Peaks **34**, **37**, and **42** were isomers with the equal molecular formula of C_22_H_28_N_2_O_4_, which were determined by the [M+H]^+^ ion at *m*/*z* 385. The MS^2^ fragments at *m*/*z* 353 and 321 were attributed to the consecutive losses of methoxyl groups. The ion at *m*/*z* 267 indicated the loss of the C_5_-side chain. By comparing their relative retention time on octadecylsilyl (ODS) column, they were deduced as isorhynchophylline, corynoxine, and rhynchophylline [26]. Peaks **27** and **31** occupied the same molecular weight of 384, corresponding to the molecular formula of C_21_H_24_N_2_O_5_. Their MS^2^ fragments at *m*/*z* 367, 351, and 335 accounting for the lost H_2_O and two additional oxygen atoms indicated an oxygenated derivative of rhynchophyllic acid. Likewise, peaks **24** and **35** were deduced as dehydro-derivatives of rhychophylline, and peak **11** was proposed as the demethylated derivative of rhychophylline [27].

Peaks **20**, **22**, **28**, **32**, and **36** had the same molecular formula of C_21_H_24_N_2_O_4_, with a CH_2_ less than corynoxeine. The MS^2^ fragmentation from *m/z* 369 to 337 verified the presence of an OMe group. The abovementioned features pointed to the demethyl corynoxeine or its isomer. The decarbonylation and decarboxylation neutral losses of 28 Da and 46 Da were proved by the ions at *m*/z 309 and 291. By retrieving the corynoxine-type alkaloids isolated from this genus, the de-methyl derivates of corynoxeine, cisocorynoxeine (**20**), 18,19-dehydrocorynoxinic acid (**22**), 18,19-dehydrocorynoxinic acid B (**28**), demethylcorynoxeine (**32**), and demethylisocorynoxeine (**36**) were proposed [28].

##### Corynanthein-Type Alkaloids

Peak **40** showed the protonated ion at *m*/*z* 355.1994, indicating the molecular formula of C_21_H_26_N_2_O_3_. The MS^2^ profiles at *m*/*z* 224.1340 (C_12_H_17_NO_3_), 212.1241 (C_11_H_17_NO_3_), and 144.0792 (C_10_H_9_N) were indicative for sitsirikine [29]. Peaks **55** and **57** were assigned as hirsuteine and hirsutine, respectively, by reason of their molecular formula (C_22_H_26_N_2_O_3_ and C_22_H_28_N_2_O_3_) and MS^2^ fragments. Peaks **48** and **53** with the same formula of C_22_H_26_N_2_O_3_ were determined to be corynantheine and geissoschizine methyl ether following their MS^2^ fragments [30]. Similarly, peaks **45** and **51** were tentatively deduced to be the dihydroxy and dihydro derivatives of corynantheine [17].

##### Ajmalicine-Type Alkaloids

Ajmalicine-type alkaloids maintain a pentacyclic heteroyohimbines framework showing similar UV absorption with corynanthein-type alkaloids. Peaks **39** and **54** were attributed with C_21_H_24_N_2_O_3_ and C_22_H_26_N_2_O_4_ with 11 double bond equivalents. The mass losses from *m/z* 352 to 321.1647 (C_20_H_20_N_2_O_2_), 222.1198 (C_12_H_15_NO_3_), 210.1126 (C_11_H_15_NO_3_), and 144.0798 (C_10_H_9_N) were in agree with ajmalicine [31]. Similarly, peak **54** was reasonably deduced to be pubescin from the MS^2^ fragments at *m/z* 223.1304 (C_15_H_14_N_2_) and 184.0878 (C_12_H_9_NO) [32].

#### Flavonoids

Flavonoids display characteristic UV absorptions at 220–280 (band II) and 300–400 (band I) nm, by which they can be easily characterized [33]. Peaks **4** and **8** with UV maximum absorption at 280 nm were designated with the molecular formula of C_30_H_26_O_12_ with 18 unsaturation degrees. Consequent MS^2^ experiment on [M+H]^+^ ion generated fragments at *m*/*z* 409 (C_22_H_16_O_8_), 301 (C_16_H_12_O_6_), and 287 (C_15_H_10_O_6_) indicating flavonoids dimers. Their relative retention time on ODS column were in accordance with procyanidin b1 (**4**) and procyanidin b2 (**8**) [34]. Peaks **5** and **10** were a pair of isomers with identical molecular formula of C_15_H_14_O_6_. The MS^2^ ion at *m*/*z* 139 (C_7_H_6_O_3_) was ascribed to the A^1,3^ retrocyclization fragment on ring C. Taking their UV absorptions at 280 nm and retention time into consideration, peaks **5** and **10** were reasonably determined as catechin (**5**) and epicatechin (**10**) [12]. Peaks **12** and **14** were isomers with the same molecular formula of C_30_H_26_O_11_, suggesting flavonoids dimers. The MS^2^ fragments at *m/z* 291.0856 (C_15_H_14_O_6_) and 273.0778 (C_15_H_12_O_5_) were attributed to fisetinidol and catechin moieties. From the above analyses, they were tentatively deduced to be fisetinidol-(4*α* → 8)-epicatechin and fisetinidol-(4*β* → 8)-epicatechin [35]. Peak **13** with a formula of C_21_H_24_O_12_ showed MS^2^ information at *m*/*z* 317.0994 (C_16_H_12_O_7_), corresponding to the loss of a C_5_ part from the *C*-glycosyl moiety. From the above analyses, this peak was defined as gallocatechol *C*-glucoside [36, 37]. Peak **16** was designed with the molecular formula of C_27_H_30_O_16_ with an additional C_6_H_10_O_4_ part than **17** (C_21_H_20_O_12_). In the MS^2^ experiment, the same fragments at *m/z* 303 in positive mode and 301 in negative mode suggested the same aglycone in **16** and **17**. By retrieving the database, they were deduced as rutin (**16**) and hyperoside (**17**) [17]. Peak **29** gave [M+H]^+^ ion at *m*/*z* 449.1068 and [M‒H]^‒^ ion at *m*/*z* 447.0939, corresponding to the molecular formula of C_21_H_20_O_11_. In the MS^2^ experiment, the diagnostic MS^2^ ions at *m*/*z* 301.0358 (C_15_H_10_O_7_) and 271.0288 (C_14_H_8_O_6_) in negative mode were indicative for the sequential loss of rhamnosyl and formaldehyde moieties. From the above analyses, this peak was deduced as quercetin 3-rhamnoside [38].

#### Chlorogenic Acids

Chlorogenic acid analogues are a type of caffeoyl quinic acids widely present in plants. In the UV spectrum, the maximum absorption at around 325 nm was due to the presence of caffeoyl group. In the MS^2^ experiment, the product ions at *m*/*z* 163 (C_9_H_6_O_3_) in positive mode and 191 (C_7_H_12_O_6_) in negative mode were indicative for caffeic acid and quinic acid moieties. In this study, four isomers, namely, neochlorogenic acid (**3**), chlorogenic acid (**6**), cryptochlorogenic acid (**7**), and isochlorogenic acid (**9**) with identical formula of C_16_H_18_O_9_ were detected and tentatively characterized by their retention time on ODS column [39]. Peak **30** was assigned with the molecular formula of C_25_H_24_O_12_ with an additional quinoyl moiety compared to chlorogenic acid. This deduction was verified by the MS^2^ ions at *m/z* 353.0882 (C_16_H_18_O_9_) and 173.0401 (C_7_H_10_O_5_) in negative mode. Thus, peak **30** was delineated as dicaffeoylquinic acid [40].

#### Triterpenoids

Peak **56** showing terminal absorption in UV spectrum was revealed with the molecular formula of C_30_H_46_O_5_. The abovementioned features were indicative for a triterpenoid. The MS^2^ fragments at *m*/*z* 469 (C_30_H_44_O_4_), 451 (C_30_H_42_O_3_), and 423 (C_29_H_42_O_2_) were in accordance with quinovic acid [41]. Peaks **49** and **52** were deduced to be diglycoside and triglycoside derivatives of quinovic acid by the additional two and three glycosyls which were verified by the sequential loss of C_6_H_10_O_5_ parts in the MS^2^ experiments. Thus, quinovic acid diglycoside and quinovic acid triglycoside were respectively determined [42].

#### Other Compounds.

Peak **1** was assigned as sucrose which was widely present in plants by the characteristic [M+K]^+^ ion at *m*/*z* 381.0792. Peak **2** had a molecular formula of C_16_H_24_O_10_ showing [M+Na]^+^ ion at *m*/*z* 399.1258 and [M‒H]^‒^ ion at *m/z* 375.1301. In the MS^2^ experiment, the loss of glycosyl was verified by the ion at *m*/*z* 215.0678 (C_13_H_10_O_3_). Thus, this peak was illustrated as loganic acid, the biosynthetic precursor of indole alkaloids [43].

### Chemical Comparison

**Fig. 2:**
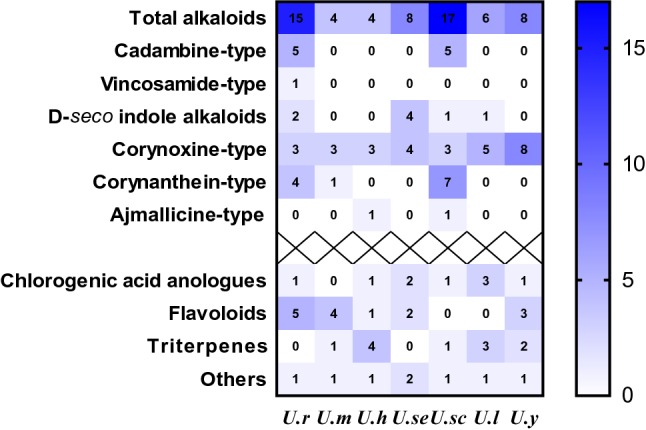
Distribution of different types of compounds among seven *Uncaria* plants

**Fig. 3:**
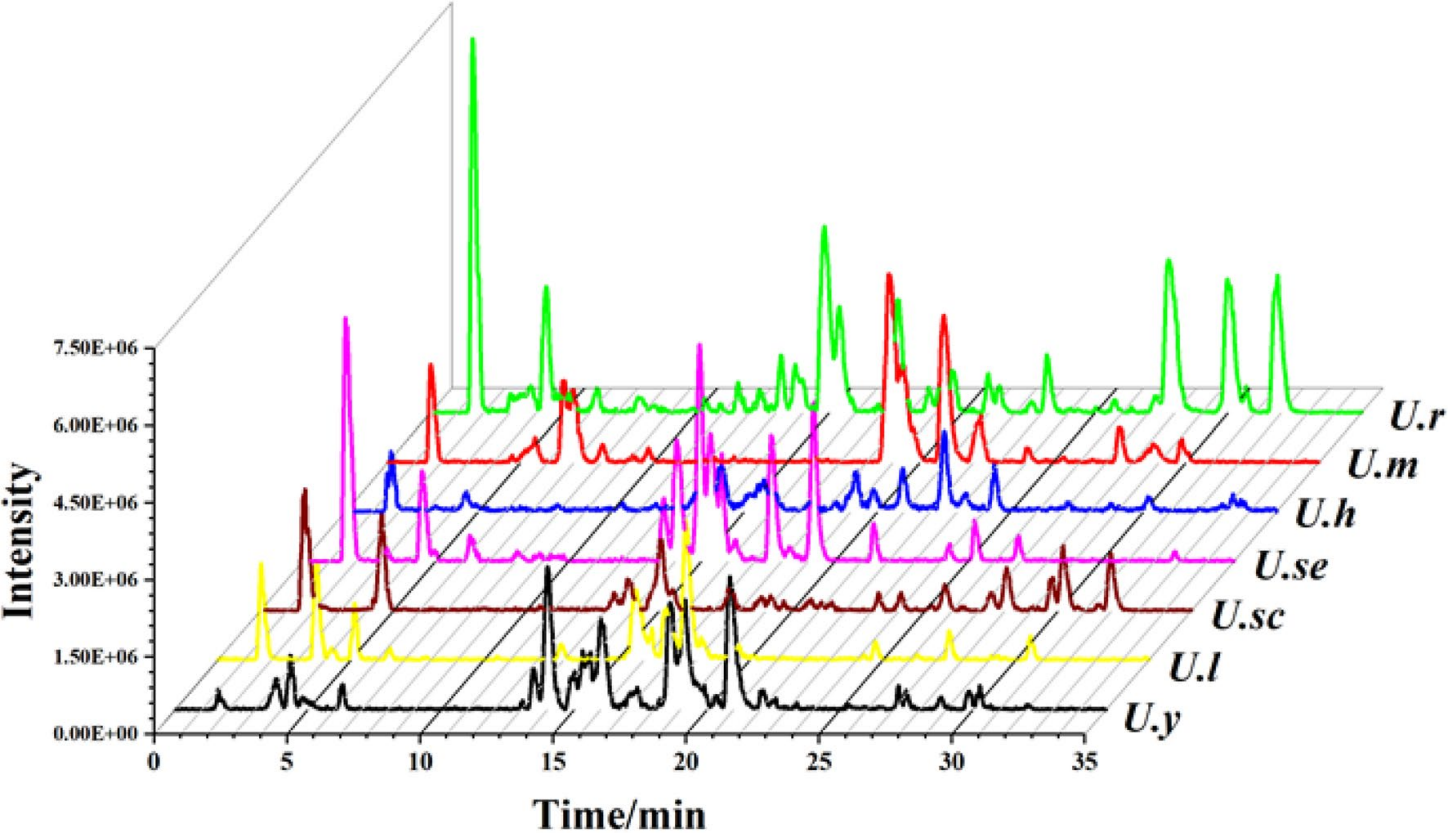
Comparison of the BPCs (positive) of seven *Uncaria* plants

As shown in Figs. 2 and 3, a temporal and spatial distribution of chemical constituents in seven *Uncaria* plants provided a visual overview of their difference. The chemical profiles of *U. rhynchophylla* and *U. scandens* were similar in terms of either indole alkaloids or other types of compounds. Indole alkaloids as the characteristic constituents were more prolific in *U. rhynchophylla* and *U. scandens* when comparing to other *Uncaria* plants. Cadambine-type and corynanthein-type alkaloids were the characteristic constituents in *U. rhynchophylla* and *U. scandens*, whereas *corynoxine-type alkaloids* were widely distributed in all the seven *Uncaria* plants. Besides alkaloids, flavonoids were another type of constituent in *Uncaria* plants, which were mainly distributed in *U. rhynchophylla, U. macrophylla*, and *U. yunnanensis*. For the triterpenoids, *U. hirsuta* and *U. laevigata* showed more prolific than other plants.

### Biological Comparison on MT_1/2_ and 5-HT_1A/2C_ Receptors

**Fig. 4:**
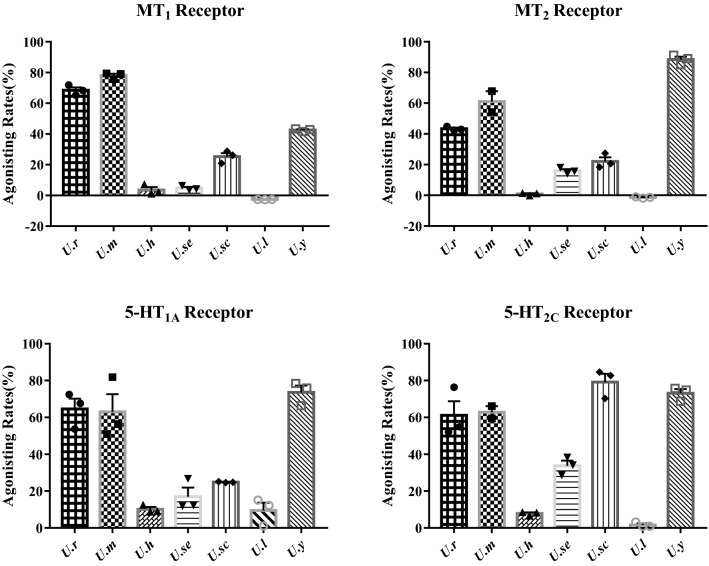
Agonistic activities of seven *Uncaria* plants on MT_1/2_ and 5-HT_1A/2C_ receptors. The agonistic activities were expressed as X ± SEM (*n* = 3), which were obtained by comparing to the positive controls, melatonin (on MT receptors) and 5-hydroxytryptamine (on 5-HT receptors)

Gou-Teng as a famous TCM are widely used for treating central nervous system (CNS) diseases in China. Therefore, four neurotransmitter receptors (MT_1_, MT_2_, 5-HT_1A_, and 5-HT_2C_) that are closely related to CNS diseases were used to evaluate the psychiatric-related effects of *Uncaria* plants. As shown in Fig. 4, three plants, *U. rhynchophylla*, *U. macrophylla*, and *U. yunnanensis* showed obviously agnostic activity on all the four receptors. As a comparison, *U. hirsuta, U. sessilifructus*, and *U. scandens* were moderate, and *U. laevigata* was less active. Specifically, *U. macrophylla* displayed the most potent activity on MT_1_ receptor with an agonistic rate of 79.0%, then followed with *U. rhynchophylla* (71.9%), *U. yunnanensis* (41.5%), and *U. scandens* (26.1%), whereas *U. hirsuta*, *U. sessilifructus*, and *U. laevigata* were inactive. For MT_2_ receptor, *U. yunnanensis* possessed the highest agonistic rate of 91.2%, and *U. macrophylla* and *U. rhynchophylla* exhibited moderate activity with agonistic rates of 54.2% and 44.8%; however, *U. scandens*, *U. sessilifructus*, *U. hirsuta*, *and U. laevigata* were weak or inactive. Similar with the MT receptors, *U. rhynchophylla*, *U. macrophylla*, and *U. yunnanensis* possessed significant activity on 5-HT_1A_ and 5-HT_2C_ receptors with agonistic rates higher that 60%. Interestingly, *U. scandens* was revealed with the highest activity on 5-HT_2C_ receptor (82.7%), almost threefold higher than 5-HT_1A_, indicating the subtype selectivity.

## Conclusion

Gou-Teng has long been recorded in ancient TCM books for the treatment of cardiovascular and mental disorders. According to the latest Chinese Pharmacopoeia, five *Uncaria* plants, *U. rhynchophylla*, *U. macrophylla*, *U. sinensis*, *U. hirsuta*, and *U. sessilifructus* are documented as the official resources of Gou-Teng. However, their chemical and biological difference as well as the discrepancy with other *Uncaria* plants are still disputed. Thus, the clinical application of Gou-Teng is confused owing to the prolific resources and morphological similarity between different species. In this investigation, seven *Uncaria* species involving four official, *U. rhynchophylla*, *U. macrophylla*, *U. hirsuta*, and *U. sessilifructus*, and three local species, *U. scandens*, *U. laevigata*, and *U. yunnanensis* were extensively compared based on LCMS and bioassay in vitro. In total, 57 constituents including 35 indole alkaloids, ten flavonoids, five triterpenoids, five chlorogenic analogues, and two other compounds were characterized based on their MS/MS patterns and UV absorptions. Cadambine-type and corynanthein-type alkaloids were exclusively present in *U. rhynchophylla* and *U. scandens*, whereas corynoxine-type alkaloids were commonly detected in all the seven *Uncaria* plants. Three *Uncaria* plants, *U. rhynchophylla*, *U. macrophylla*, and *U. yunnanensis* showed obviously agnostic activity on four receptors, suggesting their biological similarity regardless of the chemical difference. This investigation supported the synergistic effects of TCMs due to the complicated constituents and their complementarity in taking effects. This study provides valuable information for understanding the chemical and biological difference between different *Uncaria* plants and the “one-drug multi-source” theory.

## Experimental

### LCMS Analyses

LCMS analyses were performed on a Shimadzu UFLC/MS-IT-TOF apparatus (Shimadzu, Kyoto, Japan) equipped with a Welch Ultimate XB-C_18_ column (2.1 × 100 mm, *i.d*., 1.8 μm). The mobile phase for LCMS consisted of water (0.05% formic acid, A) and acetonitrile (0.05% formic acid, B) with the flow rate of 0.2 mL/min. A binary gradient elution was performed as follows: linear gradient (B%) from 10 to 35% in 35 min, and fast increased to 100% in one min and maintained for three min. Re-equilibration duration was five min between individual runs. The injection volume was 2 *μ*L for each LCMS analysis. The detailed MS parameters were set as previously reported [44]. The PDA profiles were recorded from 190 to 400 nm. The Shimadzu Composition Formula Predictor was used to speculate the molecular formula.

### Plant Materials

Plants of *Uncaria rhynchophylla* (Miq.) Miq. ex Havil. (No. 2,016,090,001), *Uncaria macrophylla* Wall. (No. 2,016,090,002), *Uncaria hirsuta* Havil. (No. 2,016,090,003), *Uncaria sessilifructus* Roxb. (No. 2,016,090,004), *Uncaria scandens* (Smith) Hutchins. (No. 2,016,090,005), *Uncaria laevigata* Wall. ex G. Don (No. 2,016,090,006), and *Uncaria yunnanensis* K. C. Hsia (No. 2,016,090,007) were collected from Xishuangbanna Dai Autonomous Prefecture of Yunnan Province in China in September 2016, and authenticated by Dr. Li-Gong Lei (Kunming Institute of Botany, CAS). Voucher specimens (No. 2,016,090,001–2,016,090,007) were deposited in the Laboratory of Antivirus and Natural Medicinal Chemistry, Kunming Institute of Botany, CAS. The hook-bearing stems were dried at room temperature and kept in amber glass flasks until extraction. The powder of each sample (2.0 g) was extracted with ethanol–water (7:3, *v/v*, 10 mL) under ultrasonic for 30 min. The extraction was filtered through a PTFE micro-porous filter (0.22 μm, Jiangsu Hanbon Science & Technology Co., Ltd.) into 2 mL screw cap vials prior to LCMS analyses.

### Agonistic Activities on MT_1/2_ and 5-HT_1A/2C_ Receptors

Bioassay for agonistic activities on melatonin and 5-hydroxytryptamine receptors was performed in accordance with the previous reports [20, 45]. In brief, HEK293 cells stably expressing human melatonin (MT_1_ and MT_2_) and 5-hydroxytryptamine (5-HT_1A_ and 5-HT_2C_) receptors were maintained in DMEM containing 10% FBS. Cells were seeded at a density of 4 × 10^4^ cells/well in pre-matrigel-coated 96-well black wall/clear bottom plates. After overnight incubation at 37 °C with 5% CO_2_, the cells were dyed with 100 μL of HDB Wash Free Fluo-8 Calcium Assay kit at 37 °C. An hour later, the cells were transferred into FlexStation3 Benchtop Multi-Mode Microplate Reader (Molecular Devices, Sunnyvale, California, United States) for bioassay. The raw data from time sequence recording were normalized as percentage responses to melatonin and 5-hydroxytryptamine as the positive controls, and analyzed to fit the four-parameter logistic equation to assess the agonistic rates.

### Statistical Analyses

All experiments were carried out in triplicate. Data were expressed as mean ± standard error of mean (Mean ± SEM). Statistical analysis was performed using GraphPad Prism 7 (GraphPad Software Inc., San Diego, CA) and Origin 2018 (OriginLab Corporation, Wellesley Hills, MA) software.

## Electronic supplementary material

Below is the link to the electronic supplementary material.

Supplementary file1 (DOCX 468 kb)
