# Supplementary material for: UFLC-PDA-MS/MS Profiling of Seven Uncaria Species Integrated with Melatonin/5-Hydroxytryptamine Receptors Agonistic Assay
Source: Nat Prod Bioprospect. 2020 Jan 13;10(1):23–36. doi: 10.1007/s13659-020-00230-8 (PMC7046893; doi:10.1007/s13659-020-00230-8)
Supplement: Supplementary file 1 — Supplementary file1 (DOCX 468 kb) [file 13659_2020_230_MOESM1_ESM.docx]

**Fig. S1**. Proposed compounds in seven *Uncaria* species based on LCMS analysis.
